# SNAP23 is selectively expressed in airway secretory cells and mediates baseline and stimulated mucin secretion

**DOI:** 10.1042/BSR20150004

**Published:** 2015-06-25

**Authors:** Binhui Ren, Zoulikha Azzegagh, Ana M. Jaramillo, Yunxiang Zhu, Ana Pardo-Saganta, Rustam Bagirzadeh, Jose R. Flores, Wei Han, Yong-jun Tang, Jing Tu, Denise M. Alanis, Christopher M. Evans, Michele Guindani, Paul A. Roche, Jayaraj Rajagopal, Jichao Chen, C. William Davis, Michael J. Tuvim, Burton F. Dickey

**Affiliations:** * Department of Pulmonary Medicine, University of Texas MD Anderson Cancer Center, Houston, TX 77030, U.S.A.; † Cystic Fibrosis Research and Treatment Center, University of North Carolina, Chapel Hill, NC 27599, U.S.A.; ‡ Center for Regenerative Medicine, Massachusetts General Hospital, Boston, MA 02114, U.S.A.; § Department of Medicine, University of Colorado School of Medicine, Aurora, CO 80045, U.S.A.; ║ Department of Biostatistics, University of Texas MD Anderson Cancer Center, Houston, TX 77030, U.S.A.; ¶ Experimental Immunology Branch, National Cancer Institute, Bethesda, MD 20892, U.S.A.

**Keywords:** 23-kDa paralogue of synaptosome-associated protein of 25 kDa (SNAP23), exocytosis, mucin, mucus, secretion

## Abstract

Airway mucin secretion is important pathophysiologically and as a model of polarized epithelial regulated exocytosis. We find the trafficking protein, SNAP23 (23-kDa paralogue of synaptosome-associated protein of 25 kDa), selectively expressed in secretory cells compared with ciliated and basal cells of airway epithelium by immunohistochemistry and FACS, suggesting that SNAP23 functions in regulated but not constitutive epithelial secretion. Heterozygous SNAP23 deletant mutant mice show spontaneous accumulation of intracellular mucin, indicating a defect in baseline secretion. However mucins are released from perfused tracheas of mutant and wild-type (WT) mice at the same rate, suggesting that increased intracellular stores balance reduced release efficiency to yield a fully compensated baseline steady state. In contrast, acute stimulated release of intracellular mucin from mutant mice is impaired whether measured by a static imaging assay 5 min after exposure to the secretagogue ATP or by kinetic analysis of mucins released from perfused tracheas during the first 10 min of ATP exposure. Together, these data indicate that increased intracellular stores cannot fully compensate for the defect in release efficiency during intense stimulation. The lungs of mutant mice develop normally and clear bacteria and instilled polystyrene beads comparable to WT mice, consistent with these functions depending on baseline secretion that is fully compensated.

## Abstract (precis)

SNAP23 (23-kDa paralogue of synaptosome-associated protein of 25 kDa) is expressed in secretory but not ciliated cells of airway epithelium, suggesting that it mediates regulated but not constitutive secretion in polarized epithelia. Baseline but not stimulated mucin secretion in heterozygous mutant mice is fully compensated by increased intracellular stores.

## INTRODUCTION

Airway mucus entraps inhaled particles and pathogens, clearing them from the lungs when ciliary action propels mucus to the pharynx to be swallowed [1,2]. The absence of airway mucus in mice results in death from microbial infection [3]. However, mucus that is excessive in amount or density cannot be cleared by ciliary action, blocking airflow and paradoxically providing a protected niche for microbial colonization. Thus, the production, secretion and maturation of mucus must be tightly controlled. Dysregulation of these processes contributes to common diseases such as asthma, cystic fibrosis and chronic bronchitis. Besides its medical significance, secretion of airway mucus serves as a useful model of polarized epithelial regulated exocytosis [4,5].

Airway mucus is formed by the synthesis, extracellular release and hydration of mucin glycoproteins by surface epithelial secretory cells and submucosal glands. Secreted mucins are large molecules with monomeric molecular masses that exceed 10^6^ Da and they further polymerize into chains and networks [1,6]. Secreted polymeric mucins are packaged dehydrated in secretory granules, and sugars comprise 50%–90% of their mass. After secretion, they absorb several 100-fold their mass of water to form mucus of the appropriate viscoelasticity to be propelled by ciliary beating [7]. The two principal polymeric mucins in the airway are Muc5ac, which is produced at low levels under healthy conditions but increases greatly in allergic inflammation or parasitic infection, and Muc5b, which is produced at substantial levels under healthy conditions and modestly increases in allergic inflammation [8,9].

Mucins are secreted both at a low baseline rate and a high stimulated rate. In healthy conditions, the airways of mice show little intracellular mucin staining because the baseline rate of secretion matches the baseline rate of synthesis so that mucin does not accumulate [9,10]. Increased mucin production in allergic inflammation exceeds the rate of baseline secretion, resulting in the accumulation of intracellular mucin and a change in cellular appearance termed ‘mucous metaplasia’. The rate of mucin secretion can be stimulated by increased levels of extracellular agonists such as ATP that activates P2Y_2_ receptors, resulting in elevation of the second messengers, diacylglycerol and calcium [4,5]. Exocytic regulatory proteins that are the targets of these second messengers in stimulated secretion are Munc13-2 (mammalian orthologue of *Caenorhabditis elegans* unc-13) that is activated by diacylglycerol and Syt2 (synaptotagmin-2) that is activated by calcium [9,11]. Baseline mucin secretion is also a regulated exocytic process as indicated by its dependency on extracellular ligands, cytoplasmic calcium and Munc13-2, though the exocytic calcium sensor in baseline mucin secretion remains unknown [4,5]. (Note that baseline regulated secretion is distinguished from constitutive secretion that transports housekeeping proteins and lipids to the cell surface.)

In contrast with progress in understanding signal transduction events in airway mucin secretion, the core exocytic machinery comprising the four-helix SNARE (soluble NSF attachment protein receptor) complex and its SM (Sec1/Munc18) protein scaffold are incompletely characterized. Previously, we found that Munc18b provides most or all scaffolding function in stimulated secretion, though only partial function in baseline secretion [12]. The single v-SNARE anchored to the vesicle membrane has been identified as VAMP8 (vesicle-associated membrane protein) [13], but the identities of the proteins contributing the three t-SNARE helices anchored to the target (plasma) membrane are unknown. In some steps of traffic, two t-SNARE helices are contributed by a single protein of the SNAP family, with one SNARE helix each at the N-terminus and C-terminus, and multiple palmitoyl groups in the central region anchoring the protein to the membrane. The best studied example of this is SNAP25 that mediates synaptic vesicle release from neuronal axons [14]. Related proteins are SNAP23, SNAP29 and SNAP47. Among these, SNAP23 is expressed ubiquitously at the level of whole organs and has been shown to function in regulated exocytosis in mast cells and platelets [15–19]. We have previously found that isoforms of regulated exocytic proteins are often shared between secretory haematopoietic and secretory airway epithelial cells [12,20,11,21]. Therefore, we examined the expression and function of SNAP23 in the airways of mice.

## MATERIAL AND METHODS

### Mice and chemicals

C57BL/6 mice were obtained from the Jackson Laboratory and bred within our colony. SNAP23 heterozygous deletant (Het) mice were crossed on to a C57BL/6 background for seven generations [22,23] and littermate controls were used in experiments comparing function between WT (wild-type) and SNAP23 Het mice. Mucin secretory function measured by PAFS (periodic acid fluorescent Schiff's reagent) staining and image analysis (see below) was indistinguishable between C57BL/6 mice and the WT progeny of SNAP23 Het mice. Muc5b null mice were generated by Christopher Evans [3]. All mice were housed in specific pathogen-free conditions and handled in accordance with the Institutional Animal Care and Use Committee of MD Anderson Cancer Center. For anaesthesia, mice were injected intraperitoneally with combination anaesthetic (ketamine 100 mg/kg, xylazine 20 mg/kg, acepromazine 3 mg/kg) or avertin (2,2,2-tribromoethanol 250 mg/kg). For killing, three times the anaesthetic dose of combination anaesthetic was used. All chemicals were obtained from Sigma–Aldrich unless otherwise specified.

### Immunohistochemistry

Lungs were inflated and fixed with 10% formalin in PBS, then embedded in paraffin as described [10]. Tissue blocks were cut into 5-μm sections, dewaxed with xylene and ethanol, washed with PBS, exposed to 3% H_2_O_2_ in 90% methanol for 30 min, then washed with PBS containing 0.05% Tween-20 (PBST) and antigens were retrieved in a heated pressure cooker for 10 min in 10 mM sodium citrate, pH 6.0. Specimens were blocked with 10% (v/v) goat serum in PBS for 1 h, washed with PBST and then labelled for 1 h with anti-SNAP23 serum raised against the peptide DRIDIANARAKKLIDS, Synaptic Systems) diluted 1:500 in PBS. Slides were then washed with PBST and incubated for 30 min at 21°C with horseradish peroxidase (HRP)-labelled goat anti-rabbit antibodies (Millipore) diluted 1:1000 in PBST, washed in PBST, developed with 3,3′-diaminobenzidene (Vector Laboratories) and counterstained with haematoxylin. For co-localization of SNAP23 with airway epithelial lineage markers, tissue blocks were cut into 2-μm sections, then stained with anti-SNAP23 serum as above; adjacent slices were stained with goat antibodies against club cell secretory protein (CCSP; 1:10000, gift of Franceso DeMayo, Baylor College of Medicine, Houston, U.S.A.) or mouse monoclonal antibodies against acetylated α-tubulin (1:200, Sigma–Aldrich) and developed with HRP-labelled secondary antibodies as above.

### Fluorescence microscopy

The lungs of 3-week-old mice were inflated with 0.5% paraformaldehyde (PFA) in PBS and fixed for 3–6 h at 21°C. For tissue sections, fixed lungs were embedded in Optimal Cutting Temperature Compound (Tissue-Tek) and cut into 10-μm sections. These were blocked in PBS with 5% normal donkey serum (Jackson ImmunoResearch) and 0.3% Triton X-100 and then incubated with the same primary antibodies used for immunohistochemistry (anti-acetylated α-tubulin 1:2500; anti-CCSP 1:2500; anti-SNAP23 1:500) diluted in the same blocking solution at 4°C overnight. Sections were then washed with PBS, incubated with secondary antibodies and DAPI (Jackson ImmunoResearch) diluted in PBS with 0.1% Triton X-100 and 0.1% Tween-20 and then washed with PBS. Images of the four fluorochromes (Figure 2) were captured on a confocal microscope (A1plus, Nikon).

For lung whole-mount immunostaining (Figure 6B), fixed lungs were dehydrated through a methanol/PBS gradient and bleached with 6% hydrogen peroxide in methanol at 4°C overnight. Bleached lungs were rehydrated through a methanol/PBS gradient and incubated with PBS with 5% normal donkey serum and 0.3% Triton X-100 and then incubated with goat anti-CCSP antibody as above and a Cy3-conjugated mouse anti-smooth muscle actin antibody (1:1000, Sigma–Aldrich) diluted in PBS with 0.3% Triton X-100 at 4°C overnight. The following day, the lungs were washed in PBS with 1% Triton X-100 and 1% Tween-20 for 6 h at 21°C and then incubated with secondary antibodies against the anti-CCSP antibody diluted in PBS with 0.3% Triton X-100 at 4°C overnight. The lungs were then washed again and fixed with 4% PFA in PBS at 21°C for 2–4 h. For optical projection tomography (OPT) microscopy, the immunostained lungs were embedded in low melting agarose (Invitrogen) and imaged on a Bioptonics 3001 OPT scanner equipped with GFP3, Texas Red and Cy5 filters at high resolution (1024 pixels). The resulting image stacks were visualized using maximal intensity projection with the Bioptonics Viewer software.

### Fluorescence-activated sorting of airway epithelial cells

A Papain Dissociation System (Worthington Biochemical Corporation) was used to dissociate airway epithelial cells from the trachea as described [24,25]. Cells from multiple mice were pooled and incubated with labelled antibodies to epithelial cell adhesion molecule (EpCAM-APC; eBiosciences) diluted 1:50 in 2.5% FBS in PBS for 30 min on ice, then sorted using a FACS Aria flow cytometer (BD Bioscences). Secretory cells were sorted from the EpCAM+ pool using labelled antibodies to SSEA-1 (SSEA-1-eFluor 650 NC; eBiosciences), ciliated cells using labelled antibodies to CD24 (CD24-PE; BD Biosciences), and basal cells using labelled lectin GSIβ4 (Griffonia simplicifolia-biotin; Sigma–Aldrich and streptavidin-APC; eBiosciences). Sorted cells were lysed with 100 μl of RIPA buffer (10 mM sodium phosphate, pH 7.2, 150 mM NaCl, 1% Triton X-100, 0.5% sodium deoxycholate, 0.1% SDS) in the presence of protease inhibitors (Roche), then frozen at −80°C for immunoblot analysis.

### Immunoblotting of SNAP23

For analysis of SNAP23 expression in sorted airway epithelial cells, lysates of equal numbers of cells were loaded in adjacent lanes for SDS/PAGE. For analysis of SNAP23 expression in the lungs of WT and mutant mice, excised lungs were homogenized in 1 ml of iced PBS using a 2-ml of ground glass tissue grinder with 0.15-mm clearance (Pyrex), diluted in PBS to 2 mg/ml, then boiled in SDS sample buffer and frozen, as described [12]. Samples of cells and lung tissue were resolved by SDS/PAGE using 11% Tris/glycine or 4%–15% Tris/HCl linear gradient gels, transferred to PVDF membranes (Millipore) and blocked with 5% non-fat milk in PBST. Blots were incubated with primary antibodies in 5% non-fat milk in PBST for 1 h, washed sequentially with PBST and PBS, then detected using secondary HRP-conjugated antibodies (Jackson ImmunoResearch) and chemiluminescence reagents (Thermo Fisher Scientific). Rabbit antiserum (1:2000) against an N-terminal peptide of SNAP23 was described previously [22]. Antibodies against acetylated α-tubulin are described under ‘Immunohistochemistry’ above. Antibodies against β-actin (rabbit monoclonal 13E5, 1:1000) or GAPDH (glyceraldehyde 3-phosphate dehydrogenase, rabbit monoclonal 14C10, 1:5000; both from Cell Signaling) were used as loading controls.

### Airway epithelial mucin content by PAFS staining and image analysis

To evaluate stimulated secretion, mucous metaplasia was induced in the airways of mice by intraperitoneal immunization followed by aerosol challenge with ovalbumin [10]. Three days after ovalbumin aerosol exposure, half of the mice in each group were exposed for 5 min to an aerosol of 100 mM ATP in 0.9% NaCl to induce mucin secretion and then sacrificed after 20 min. To evaluate baseline secretion, mice were not exposed to ovalbumin or ATP (naive state). For both sets of mice, lungs were harvested, fixed in 10% formalin, embedded in paraffin and sectioned as described above for ‘Immunohistochemistry’. Slides were deparaffinized, rehydrated and then stained with PAFS as described previously [10]. For the quantification of intracellular mucin, data are presented as the epithelial mucin volume density, derived as described [10,11,26]. Images were acquired and analysed by investigators blinded to mouse genotype and treatment.

### Western blotting of mucins in lung lysates

Lungs were harvested and frozen, then later homogenized in 1 ml of 6 M guanidinium buffer containing protease inhibitors and incubated overnight at 4°C and centrifuged as described [26]. Supernatants were dialysed overnight at 4°C against 6 M urea buffer in Slide-A-Lyzer 10K MWCO 3 ml Dialysis Cassettes (Thermo Fisher Scientific), reduced with DTT and then alkylated with iodoacetamide, electrophoresed through 1% agarose gels, then transferred by vacuum to nitrocellulose membranes (Whatman). Membranes were washed in PBS, then pre-incubated with PBST before probing with lectin UEA-1 in PBST (1:200 dilution of a 1 mg/ml solution, Sigma-Aldrich) to detect fucosylated Muc5ac [26] or blocked with 5% non-fat milk in PBST before probing with monoclonal antibody MDA-3E1 raised by us against peptide TTCQPQCQWTKWIDVDYPSS in PBST (1:3000) to detect Muc5b. To confirm the identity of the band detected by UEA-1 as Muc5ac, parallel blots were probed with monoclonal antibody 45M1 (1:500, Thermo Fisher Scientific) and displayed immunoreactivity at the same relative mobility as the binding of labelled UEA1. To confirm the specificity of MDA-3E1 for Muc5b by immunohistochemical staining, tissue was processed as for SNAP23 staining, above, and probed with MDA-3E1 (1:1000).

### Secreted mucins from perfused tracheas

Mucous metaplasia of tracheal epithelial cells was induced by the instillation of IL-13 (40 ng/μl IL-13, 2 μl/g body weight; BioLegend) into the oropharynx of mice under isoflurane anaesthesia. The mice were administered IL-13 on two successive days and on the third day were killed and the tracheas harvested by dissection. The tracheas were cannulated at their proximal ends, mounted vertically in a perfusion chamber as described previously [9,27] and perfused with DMEM (Dulbecco's modified Eagle's medium; 4.5 g/ml glucose) culture medium at 10 μl/min for a 2 h equilibration period. After equilibration, 50 μl fractions were collected every 5 min for a 30 min baseline period, then the perfusate was switched to one containing ATPγS (100 μM) and fractions were collected for another 30 min. The fractions were assessed for released mucins by ELISA using a mucin ‘subunit’ antibody as described previously [9,27].

### Mucociliary clearance and bacterial colonization

Mucociliary clearance was measured as the elimination of fluorescent microspheres from the lungs over time, as described [3]. Briefly, 5×10^5^ yellow-green fluorescent carboxylate-modified 2 μm diameter polystyrene microspheres (Fluospheres, Life Technologies) in 25 μl of PBS were instilled intratracheally into anaesthetized mice by a transoral route using a 200 μl gel-loading pipette tip. Mice were then killed by exsanguination immediately after microsphere instillation or 15 min later. Lungs and trachea (up to but not including the larynx) were excised, placed in vials containing PBS/0.1% Tween-20 and 1-mm diameter glass beads and homogenized by agitation at 4800 oscillations/min for 10 s using a Mini-Beadbeater-1 (Bio-Spec Products). Homogenized material was placed on a haemacytometer and beads in the lungs were quantified using epifluorescence microscopic imaging. Baseline deposition was assessed by averaging the numbers of microspheres detected in the lungs in mice killed immediately after installation. Fractional clearance was calculated by subtracting the ratio of the numbers of beads remaining in the lungs at 15 min over the average at time zero for each genotype, subtracting the ratio from 1 and multiplying the difference by 100 to express the result as percentage cleared. To measure bacterial counts, the lungs were removed from killed mice by sterile dissection and then homogenized in 1 ml PBS using a 2-ml ground glass tissue grinder as above. The homogenates were serially diluted on to tryptic soy agar plates (Becton, Dickinson), incubated at 37°C for 16 h and bacterial colonies were counted.

### Lung mechanics

Respiratory resistance was analysed using a flexiVent (Scireq) forced oscillation ventilator system for measurement of lung mechanics. Mice were anaesthetized with urethane (3 mg/g by intraperitoneal injection, a dose sufficient for 2 h of sedation though experiments last less than 30 min) and paralysed with succinylcholine chloride (5 mg by intraperitoneal injection followed by continuous intraperitoneal infusion at 20 μg/g·min). Mice were tracheostomized with a blunt beveled 18-gauge Luer-Stub adapter (Becton, Dickinson) and ventilated at 150 breaths/min, 10 μl/g, against 2–3 cm H_2_O positive end-expiratory pressure. Respiratory resistance was assessed at baseline and in response to three incremental doses of aerosolized methacholine (MCh; 3, 10 and 30 mg/ml) administered by an in-line ultrasonic nebulizer. Total respiratory resistance was calculated by averaging five values measured for each dose of MCh for each mouse.

### Statistical analyses

Data are presented as means ± S.E.M. and statistical analysis was performed using Student's *t*test (SPSS Statistics, version 16.0). *P*<0.05 was considered statistically significant.

## RESULTS

### Selective expression of SNAP23 in lung secretory cells

SNAP23 is ubiquitously expressed at the level of whole organs [28,29]. To determine the cellular expression of SNAP23 among the heterogeneous cell types of the lungs, we performed an immunohistochemical analysis using SNAP23-specific antiserum (Figure 1). Mouse lung sections exposed to control non-immune serum showed faint brown diaminobenzidine precipitate throughout the tissue section, consistent with non-specific background staining (Figure 1A, left). Lung sections exposed to α-SNAP23 serum showed intense brown staining in a subset of airway epithelial cells (Figure 1A, centre). In addition, a thin line of stain was visible at the lumenal surface of adjacent blood vessels, consistent with the reported expression of SNAP23 in endothelial cells [30,31]. Examination of the airway at higher magnification (Figure 1A, right) revealed that cytoplasmic α-SNAP23 staining was present in cells with an apical domed morphology and lacking cilia, consistent with the structure of secretory (club) cells (arrowheads), but faint or absent from interpolated ciliated cells (arrows). In alveoli surrounding the airways, stain was present in rounded cells suggestive of the morphology of type 2 alveolar epithelial cells that secrete pulmonary surfactant (open arrowhead), consistent with a prior report of the inhibition of surfactant secretion from permeabilized type 2 cells by α-SNAP23 antibodies [32].

**Figure 1:**
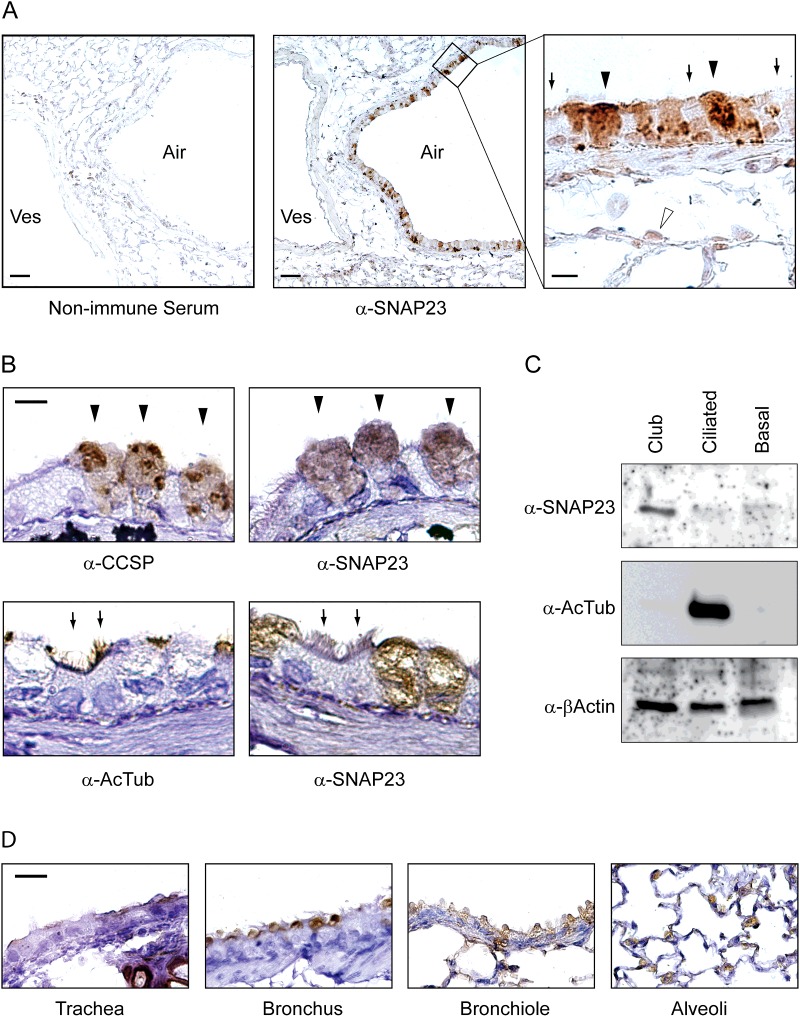
Expression of SNAP23 in the lungs of mice (**A**) Sections of mouse lung were incubated with non-immune rabbit serum (left) or rabbit serum reactive to SNAP23 (centre), then labelled with HRP-conjugated secondary antibodies. Lumens of airways (Air) and blood vessels (Ves) are indicated. A high magnification image of a portion of the centre image is shown on the right, with closed arrowheads pointing to stained club cells, arrows pointing to unstained ciliated cells and an open arrowhead pointing to a type 2 alveolar epithelial cell. Scale bar in the left and centre images=100 μm and in the right image=10 μm. (**B**) Adjacent thin sections of mouse airways were labelled with antibodies against CCSP or SNAP23 (top row) and arrowheads point to the same three club cells stained in each image. Other adjacent sections were labelled with antibodies against acetlylated α-tubulin (AcTub) or SNAP23 (bottom row) and arrows heads point to the same two ciliated cells stained with acetylated tubulin but not with SNAP23 antibodies. Scale bar=10 μm. (**C**) Mouse tracheal epithelial cells were separated by FACS using lineage-specific surface markers, then equal numbers of club, ciliated and basal cells were Western blotted and probed with antibodies to SNAP23, acetylated α-tubulin as a lineage probe and β-actin as a loading control. (**D**) Sections of airways at multiple levels from the trachea to alveoli were probed with antibodies against SNAP23. Scale bar=20 μm.

To further establish the identity of airway epithelial cells stained with α-SNAP23 antibodies, adjacent tissue sections were labelled with antibodies that mark lineages (Figure 1B). Domed cells at the epithelial surface expressing CCSP, which marks airway secretory cells, stained intensely with α-SNAP23 antibodies (Figure 1B, top, arrowheads). In contrast, cells with ciliary tufts expressing acetylated tubulin, which marks ciliated cells, were not stained with α-SNAP23 antibodies (Figure 1B, bottom, arrows). Similarly, by immunofluorescence microscopy (Figure 2), SNAP23 staining (red) was seen in epithelial cells that also stained for CCSP (blue) but not for acetylated tubulin (green). SNAP23 staining was noted to be punctate and at least partly intracellular, consistent with prior reports of SNAP23 localization to intracellular compartments [16,33–35]. Staining was predominantly in the apical region of secretory cells, but some SNAP23 punctae were localized basolaterally (Figure 2), also consistent with prior reports [33,36,37]. Cells in the submucosa, presumably mesenchymal cells such as smooth muscle cells and fibroblasts, were not stained with α-SNAP23 antibodies (Figure 2).

**Figure 2:**
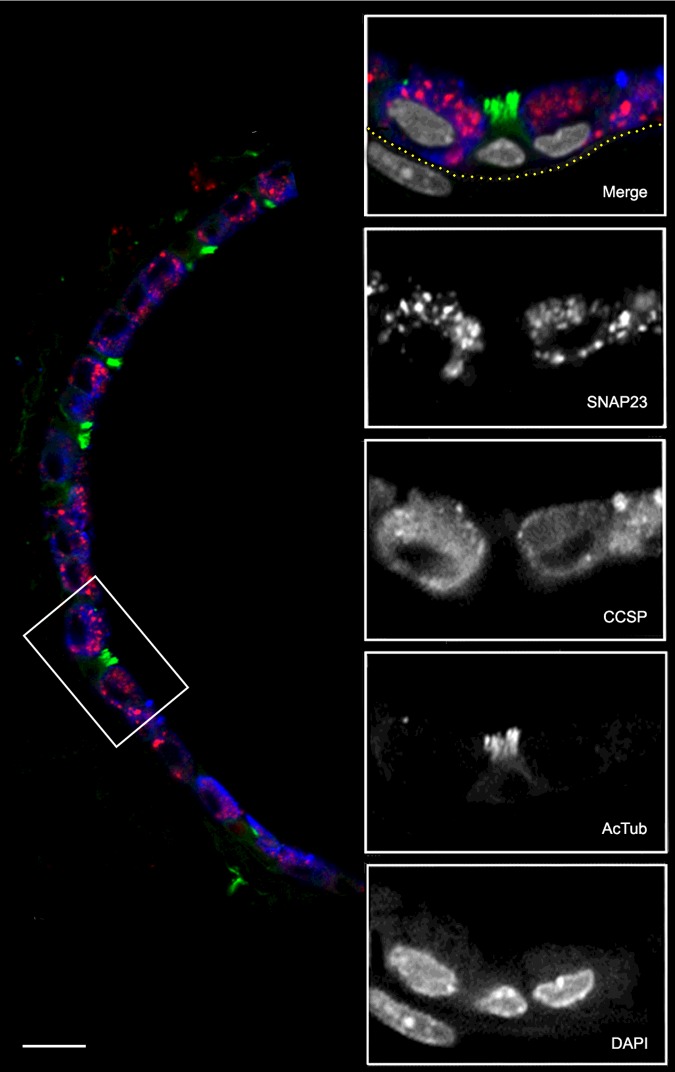
Co-localization of SNAP23 with airway epithelial lineage markers A section of mouse lung was probed with primary antibodies from different species and then labelled with species-specific fluorescent secondary antibodies. The low-magnification image (left) shows the epithelium of a small airway with SNAP23 stained red, CCSP stained blue and AcTub stained green. The boxed area is shown at higher magnification with DAPI-labelled nuclei stained grey in the merged image and with each fluorochrome shown separately in grey scale below. The sub-epithelial basement membrane was identified by auto-fluorescence in the green channel and its position is indicated by the yellow dotted line. The nucleus in the submucosa probably belongs to a smooth muscle cell based on its elongated shape and proximity to the epithelium. Scale bar for low magnification image=20 μm and for high magnification images=10 μm.

To confirm the selective expression of SNAP23 in secretory airway epithelial cells, epithelial cell lineages from disaggregated mouse lungs purified by FACS were probed by Western blotting. Airway secretory cell lysates stained intensely at 23 kDa with α-SNAP23 antibodies, whereas ciliated and basal cell lysates did not show detectable staining (Figure 1C). Probing the same blots with antibodies specific for β-actin showed similar staining of all three cell types, confirming comparable loading of the blot lanes, and antibodies specific for acetylated tubulin showed intense staining of ciliated cell lysates, confirming the efficacy of ciliated cell isolation.

To examine the expression of SNAP23 in epithelial cells along the full length of the airspaces of the lungs, tissue sections at multiple levels were examined immunohistochemically (Figure 1D). SNAP23 staining was seen in secretory but not ciliated cells of the trachea and the proximal (bronchial) and distal (bronchiolar) conducting airways (Figure 1D, three left panels). At the level of alveoli, staining was seen in rounded cells at interstices, consistent with the morphology and position of type 2 cells, but not in flattened cells of alveolar walls consistent with non-secretory type 1 cells (Figure 1D, right). Together, these results indicate that SNAP23 is highly expressed in secretory epithelial cells throughout the conducting airways and alveoli, but expressed only at low levels or not at all in airway ciliated and basal cells and alveolar type 1 cells.

### Defects in baseline and stimulated airway mucin secretion in SNAP23 mutant mice

The selective expression of SNAP23 in airway secretory cells suggested a role in airway mucin secretion. To evaluate this possibility, we studied mutant mice with deletion of the first coding exon of SNAP23 [22,23]. Null mice are not viable, so we used Het deletant mice. Airway mucins are secreted at a low baseline rate and a high stimulated rate that can be uncovered by distinct mutations [9,11]. Mucin secretion was analysed by three techniques: measurement of intracellular mucin stores by image analysis of bronchial airways stained histochemically with PAFS [26], measurement of intracellular mucin stores by Western blotting [26], and measurement of extracellular mucins secreted from perfused tracheas by ELISA [9].

By PAFS staining, intracellular mucin was not visible in naïve (uninflamed) WT mice (Figure 3A) [9,10,26]. In contrast, the airways of naive SNAP23 Het mice showed spontaneous accumulation of intracellular mucin (Figure 3A), indicating a defect in baseline secretion [26]. By image analysis, intracellular mucin was 3–4-fold greater in Het than in WT mice (Figure 3A). To more precisely measure intracellular mucin content, Western blots of lung homogenates were probed with a specific monoclonal antibody we raised against the secreted mucin, Muc5b (Figures 4A and 4B). The specificity of the α-Muc5b antibody was demonstrated by staining of airway secretory cells but not ciliated cells of WT mice (Figure 4A) and the lack of any staining of the airways of Muc5b KO (homozygous gene deletant) mice (Figure 4B). By immunoblotting, Muc5b was increased 3-4-fold in naïve lungs of Het mice compared with WT (Figure 4C), similar to what was observed by image analysis of PAFS staining (Figure 3A). By lectin blotting as previously described [26], Muc5ac was not detectable (Figure 4D), consistent with the known low expression of Muc5ac in lungs of naive mice [3,26]. The quantification of intracellular mucin by Western blotting is shown in Supplementary Figures S1 and S2.

**Figure 3:**
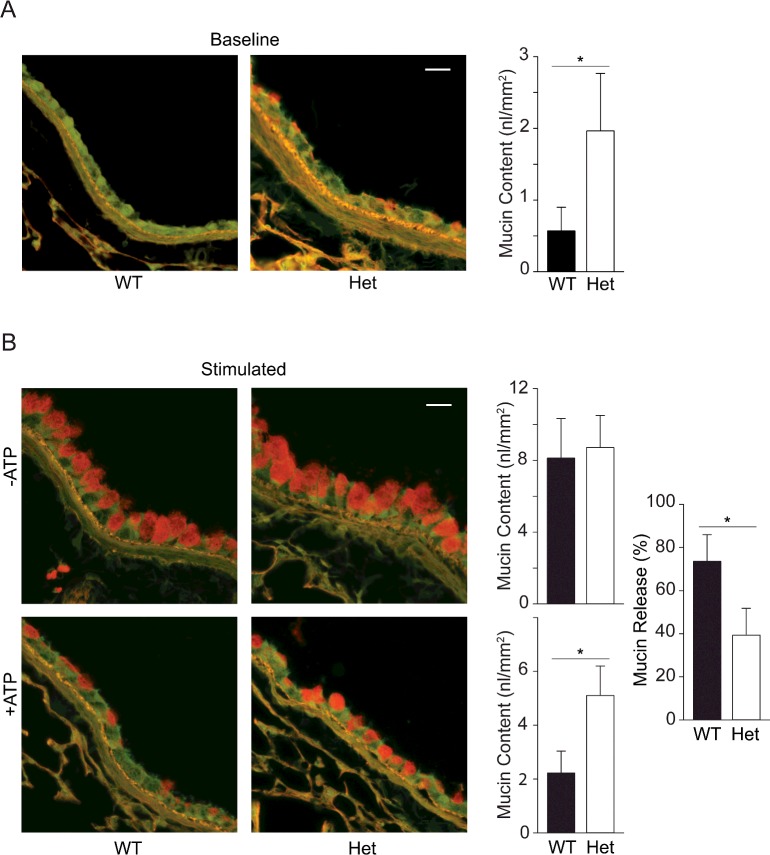
Steady state mucin secretory function in WT and SNAP23 Het mice (**A**) Baseline secretory function was examined by PAFS staining of bronchial airways of untreated WT and Het mice. Spontaneously accumulated intracellular mucin stains red and is quantified in the bar graph by analysis of images from two airway sections each from ≥ seven mice per group. *P<0.05. Scale bar=20 μm. (**B**) Stimulated secretory function was examined by PAFS staining of bronchial airways of mice with mucous metaplasia induced by ovalbumin sensitization and challenge, then not further treated (top row) or treated with an aerosol of 100 mM ATP to induce acute mucin secretion (bottom row). Intracellular mucin content is quantified in the adjacent bar graphs and the mucin content in mice treated with ATP as a percentage of that in untreated mice is shown at far right. Results are the weighted means ± S.E.M. of ≥ two separate experiments with a total of ≥ 12 mice per group, *P<0.05.

**Figure 4:**
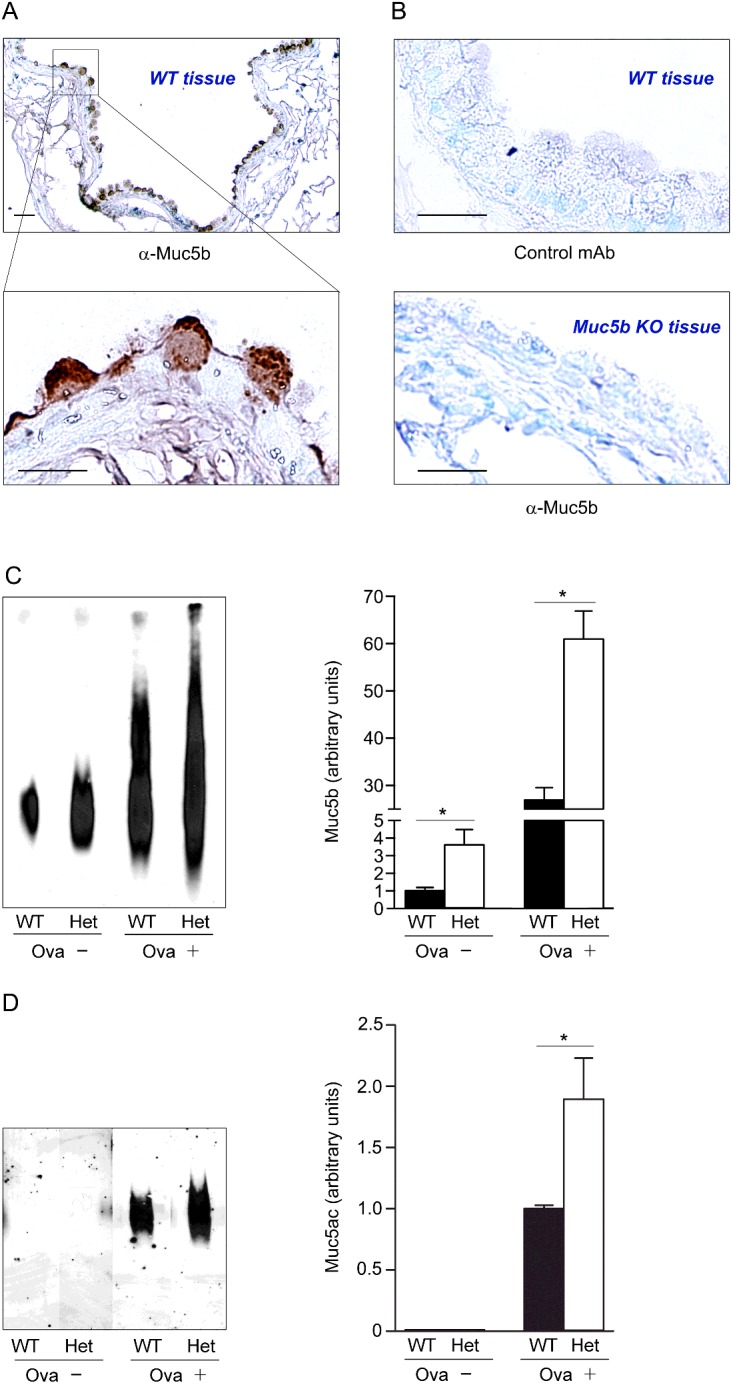
Measurement of Muc5ac and Muc5b in lungs of WT and SNAP23 Het mice (**A**) Monoclonal antibody MDA-3E1 generated against a peptide in Muc5b stains granules in naive airway secretory cells. Scale bar=20 μm in low magnification image and 10 μm in high magnification image. (**B**) There is no brown staining with an isotype control monoclonal antibody (top) or with MDA-3E1 in Muc5b KO mice (bottom). (**C**) Immunoblot of lung lysates from WT and SNAP23 Het mice, without airway inflammation (Ova−) or with allergic airway inflammation (Ova+), probed with antibody MDA-3E1 to detect Muc5b. Densitometric analysis of three similar blots are plotted at right showing means ± S.E.M., *P<0.05. (**D**) Western blot of lung lysates from WT and SNAP23 Het mice, with or without airway inflammation as in (**C**), probed with UEA1 lectin to detect Muc5ac. Densitometric analysis of three similar blots is plotted at right, as for (**C**).

To assess stimulated secretion, mucous metaplasia (i.e., increased mucin production) was first induced by allergic inflammation using ovalbumin sensitization and challenge, then mice were exposed by aerosol to the strong secretagogue ATP [26]. The airways of SNAP23 Het mice with mucous metaplasia did not show measurably higher mucin content by PAFS staining than the airways of WT mice (Figure 3B, top row), probably because the defect in baseline secretion results in only modest additional intracellular mucin accumulation relative to the large amount of newly synthesized mucin that saturates the fluorescence image. Consistent with this interpretation, Muc5ac and Muc5b glycoprotein levels were both increased ~2-fold during mucous metaplasia in Het mice compared with WT by Western blotting (Figures 4C and 4D). After the stimulation of mucin secretion by exposure to an ATP aerosol, Het mice appeared to release only half as much intracellular mucin as WT mice by PAFS staining (Figure 3B, bottom row), indicating a defect in stimulated secretion, though image saturation of the unstimulated airways precludes the calculation of fractional mucin release. The stimulated loss of intracellular mucin content cannot be measured by Western blotting because the rapid release of large amounts of mucin from metaplastic epithelium results in mucus impaction in the airways so that it is not cleared from the lungs, characteristic of the pathophysiology of asthma [1]. Therefore the defect in stimulated mucin secretion could not be precisely quantified by either of the static assays (image analysis and Western blotting).

Measurement of secreted mucins in isolated perfused metaplastic tracheas showed no detectable difference between Het and WT mice in baseline secretion (-25 through 0 min; Figure 5A). This result was surprising in view of the baseline secretion defect detected by PAFS staining of naive airways (Figure 3A) and by Western blotting of both naive and metaplastic airways (Figures 4C and 4D). A possible explanation is that the reduced efficiency of the exocytic machinery in SNAP23 Het mice is balanced by greater intracellular mucin stores so that secreted mucins reach the same steady state baseline rate in Het and WT mice. This was further explored by analysing stimulated secretion, which showed complex kinetics (Figure 5). During the initial 10 min of stimulation with ATP, WT mice secreted 32% more mucin than Het mice, but during the subsequent 45 min, Het mice secreted 64% more, with Het mice secreting 11% more during the full 55 min period of observation. This is consistent with the interpretation of counteracting effects of reduced exocytic efficiency and increased intracellular mucin stores in SNAP23 Het mice, such that the reduced exocytic efficiency of Het mice dominates during the acute phase of stimulated secretion since WT but not Het mice almost completely empty intracellular stores within 5 min (Figure 3B). Conversely, the increased intracellular stores of Het mice dominate during chronic stimulated secretion after intracellular stores become depleted in WT mice (Figure 3B). Thus, the effect of deficiency of SNAP23 on mucin secretion measured in the extracellular medium is inapparent at baseline and during stimulation is better revealed by kinetic than aggregate analysis (Figure 5).

**Figure 5:**
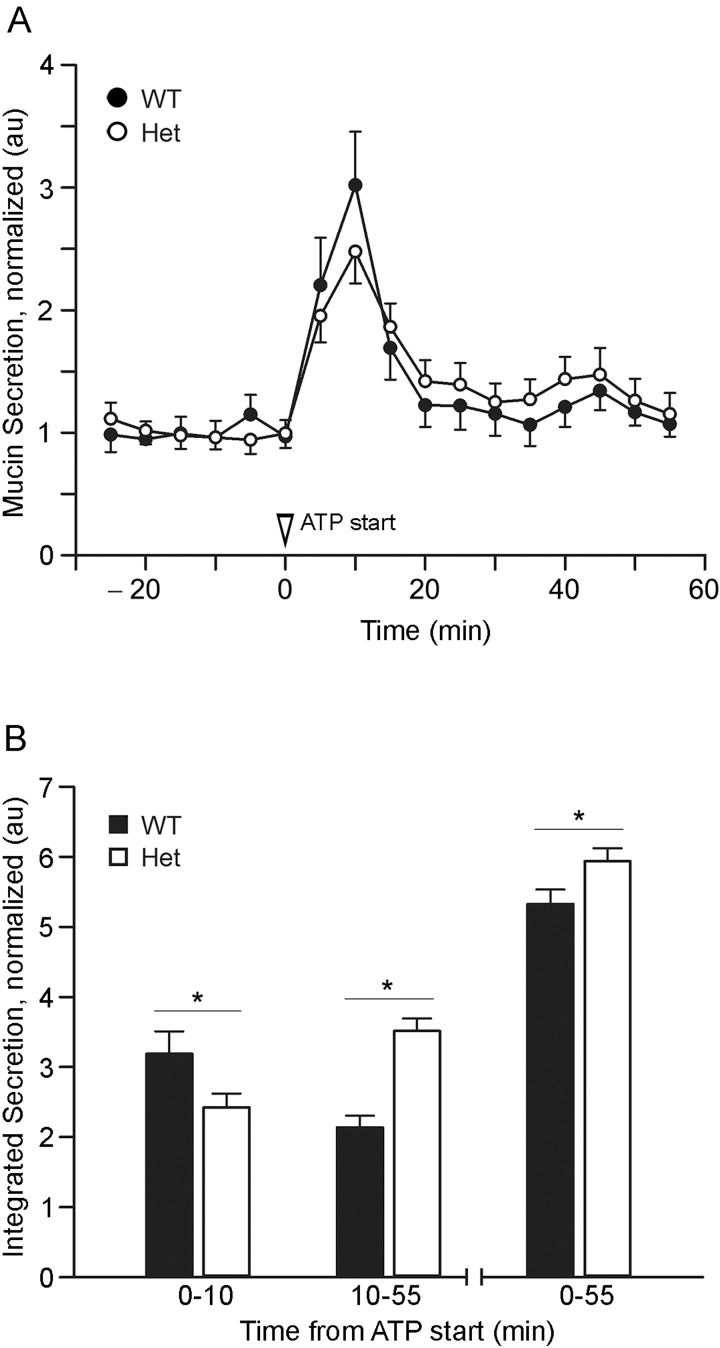
Kinetic mucin secretory function in WT and SNAP23 Het mice (**A**) Mucous metaplasia was induced by intrapharyngeal instillation of interleukin (IL)-13, then tracheas were excised and perfused with isotonic cell culture medium; secreted mucins in the perfusate were measured every 5 min. At time 0, 100 μM ATPγS was added to the perfusate. Results are the weighted means ± S.E.M. of ≥ two separate experiments with a total of ≥ eight mice per group. (**B**) Total mucins secreted during the early (0–10 min), late (10–55 min) or entire (0–55 min) periods of stimulation with ATPγS are compared between WT and Het mice, *P<0.05.

### Lack of defects in lung structure or clearance function in SNAP23 mutant mice

In view of the defects in baseline and stimulated mucin secretion in Het mutant mice, the structure and clearance function of mutant mouse airways were examined. To confirm that SNAP23 expression is proportionally reduced in the lungs of SNAP23 Het mice as it is in the brain [22], we immunoblotted lung lysates and found SNAP23 expression reduced by approximately 50% (Figure 6A). In view of the large increase in secretory product during mucous metaplasia and the fact that SNAP23 expression is limiting for stimulated secretory function (Figures 3 and 5), we wondered whether SNAP23 expression would increase during mucous metaplasia. We found no difference in the level of SNAP23 protein by immunoblot analysis of lung lysates in WT mice with and without mucous metaplasia (Figure 6B).

**Figure 6:**
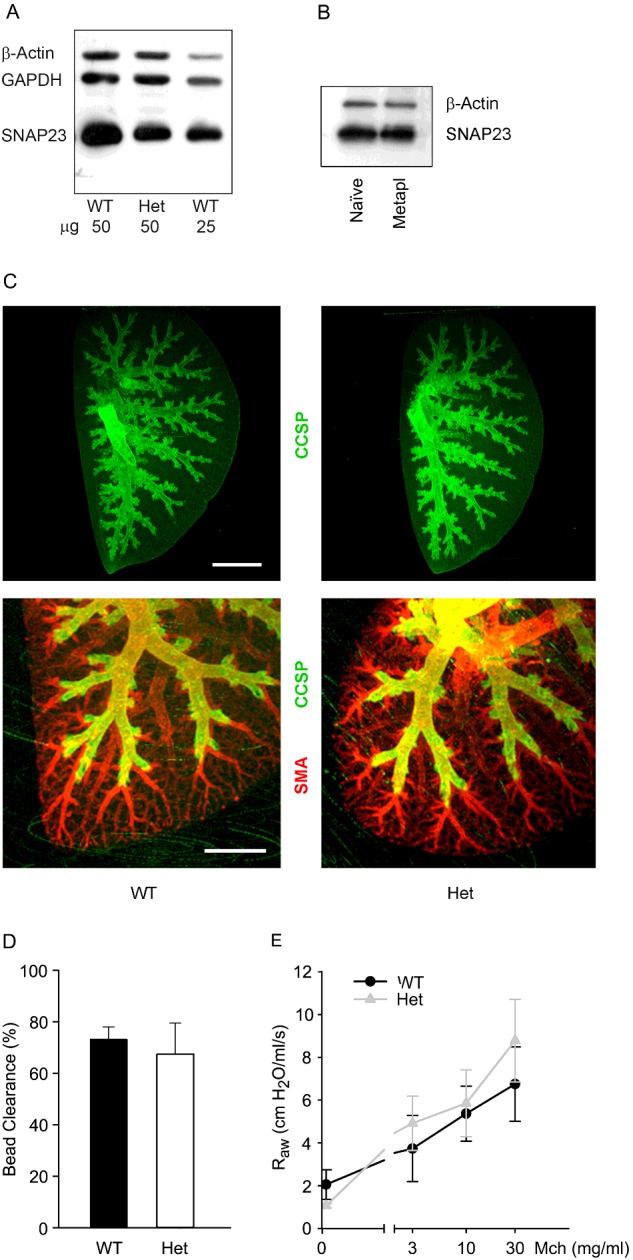
Structure and clearance function of the lungs of WT and SNAP23 Het mice (**A**) Western blots of lung homogenates from adult WT and Het mice were probed with antibodies against SNAP23. Antibodies against β-actin and GAPDH were used as loading controls. (**B**) Western blots of lung homogenates from WT mice, with or without mucous metaplasia, were probed with antibodies against SNAP23. Antibodies against β-actin were used as a loading control. (**C**) The branching structure of airways from 3-week-old WT and Het mice was revealed by whole mount immunostaining using antibodies against CCSP to mark airway secretory cells and against smooth muscle actin (SMA) to mark arterioles. Scale bar in top image=2 mm and in bottom image=1 mm. (**D**) Fluorescent 2 μm polystyrene microspheres were instilled into the lungs of adult WT and Het mice and the percentage of microspheres cleared from the lungs in 15 min was measured as a percentage of those instilled. Results are the weighted means ± S.E.M. of ≥ two separate experiments with a total of ≥ four mice per group. (**E**) Total respiratory resistance during mechanical ventilation with MCh aerosol challenge. Results are the weighted means ± S.E.M. of ≥ two separate experiments with a total of ≥ four mice per group.

There were no obvious defects in gross lung morphology or the microscopic structure of inflated, fixed and sectioned lungs of SNAP23 Het mice (result not shown). Lung structure was further examined using lung whole-mount immunostaining with antibodies against CCSP and smooth muscle actin. There were no apparent differences in the branching morphology or lumenal diameter of the airway tree or the vascular tree between WT and SNAP23 Het mice (Figure 6C). Mucociliary clearance function was first examined by measurement of the fraction of polystyrene beads instilled by tracheal insufflation that were cleared from the lungs by 15 min (Figure 6D). There was no significant difference between the fraction cleared in WT mice (72±4%) and the fraction cleared in SNAP23 Het mice (67±11%). Next, the degree of bacterial colonization of the lungs of adult mice was assessed by culture. There was no significant difference in the number of bacterial colonies cultured from the lungs of WT (430±83 colonies) and SNAP23 Het (433±160 colonies) mice (result not shown). Thus, there were no apparent abnormalities in lung structure or clearance function in SNAP23 Het mice despite the defects in mucin secretion, suggesting a threshold above which partial mucin secretory function is sufficient to support normal lung structure and clearance function.

In view of the blunting of acute stimulated mucin secretion in SNAP23 Het mice (Figures 3A and 4), we examined whether SNAP23 Het mice would be protected against the airflow obstruction that occurs when WT mice with mucous metaplasia and smooth muscle hyper-responsiveness from allergic inflammation are exposed to a ligand that causes mucin secretion and smooth muscle contraction [3,38]. Allergic airway inflammation was induced by ovalbumin immunization and challenge and mice were then anaesthetized, mechanically ventilated and exposed to increasing doses of aerosolized MCh. WT mice without allergic inflammation showed a maximal respiratory resistance of 2–3 cm H_2_O·s·ml^−1^ (result not shown), whereas WT mice with allergic inflammation showed a maximal respiratory resistance of 5–9 cm H_2_O·s·ml^−1^ (Figure 6E, circles). There was no reduction of the rise in respiratory resistance from MCh in SNAP23 Het mice with allergic inflammation (Figure 6E, triangles).

## DISCUSSION

### Expression of SNAP23 in lung epithelial secretory cells

SNAP23 transcripts and protein are ubiquitously expressed at the level of whole organs in mice [28,29]. However, to our knowledge, the differential expression of SNAP23 within various cell types within an organ has not previously been examined. We found SNAP23 highly expressed in secretory epithelial cells of the conducting airways and the alveoli (Figures 1 and 2), but not in non-secretory epithelial cells or mesenchymal cells. This suggests that SNAP23 might serve as a useful marker of the apical secretory lineage in airway epithelial cells, as we have previously found for the exocytic proteins Rab3D, Syt2 and Munc18b [10–12]. The selective expression of SNAP23 in lung epithelial cells specialized for apical secretion suggests that it functions exclusively in regulated exocytosis, similar to the role of SNAP25 in neurons [10–12,14]. Our studies do not rule out the possibility that SNAP23 also participates in constitutive secretion of housekeeping proteins in epithelial secretory cells, nor that low levels of SNAP23 below the limits of our methods of detection mediate constitutive secretion in non-secretory epithelial cells. However, SNAP23 has been found to function only in regulated exocytic processes in other cell types [15,16,19,32,39] and knockdown of SNAP23 in HeLa cells did not impair constitutive exocytosis [40]. Thus, it seems more likely that another protein(s) supplies the non-Syntaxin t-SNAREs for constitutive exocytosis in both secretory and non-secretory cells, such as SNAP29 [41–43].

The punctate intracellular localization of much of the SNAP23 staining in airway secretory cells (Figure 2) is somewhat surprising in view of its apparent role in secretion, where it is expected to participate in t-SNARE complexes in the plasma membrane. However, prior studies have also localized SNAP23 to intracellular compartments as well as the plasma membrane [16,33–35]. Of particular interest is a study of SNAP24, the *Drosophila* orthologue of SNAP23 [35]. Salivary glands in third instar larvae synthesize glycoproteins termed glue proteins that are homologous to mammalian mucins, are stored in secretory granules and at the onset of pupation are secreted into the gland lumen to fix the puparium to a substrate. Early in salivary gland development, SNAP24 is localized to the surface of small secretory granules. With maturation, the granules enlarge due to homotypic fusion and SNAP24 continues to localize on granule surfaces. At the onset of secretion, granules fuse with each other and with the apical membrane in compound exocytosis, suggesting that only a small proportion of SNAP24 is initially localized at the plasma membrane during stimulated exocytosis with the remainder translocated from fusing granules. Redistribution of SNAP23 has also been described during mast cell exocytosis [16]. In dendritic cells, SNAP23 mediates the fusion of recycling endosomes containing MHC-I with phagosomes containing microbial components [34], rather than mediating exocytosis. Thus, roles for SNAP23/24 have been reported both for intracellular and exocytic fusion events.

### SNAP23 mediates baseline and stimulated airway mucin secretion

Airway mucin is secreted at a low baseline rate and a high stimulated rate. Both modes of secretion are regulated as indicated by their dependency on extracellular ligands, cytoplasmic calcium and the exocytic protein Munc13-2 that is the target of second messengers [4,5]. Some exocytic proteins participate exclusively in one mode of mucin secretion, such as the calcium sensor Syt2 that is required for stimulated but not baseline secretion [11]. We find that SNAP23 mediates both baseline and stimulated airway mucin secretion as revealed by multiple assays as follows.

For baseline secretion, static assays (image analysis in Figure 3 and Western blotting in Figure 4) show a clear defect in SNAP23 Het mice, whereas the kinetic assay of released mucin shows no difference between WT and Het mice (Figure 5A, time period −25–0 min). The simplest interpretation of these data is that increased intracellular mucin stores in Het mice compensate for decreased release efficiency, with mucin synthesis remaining constant. In fact, this outcome is inevitable unless progressive mucin accumulation was to lead to a reduction in mucin synthesis, but this is ruled out by the preservation of steady state release in metaplastic airways (Figure 5A). This can be conceptualized as a hydrostatic steady state, such as a bucket being filled with water at a constant rate (mucin synthesis) and draining from an outlet (mucin secretion) at the same rate (Figure 7). If the outlet is made smaller (SNAP23 mutant), outflow does not change because a taller column of water (mucin store) exerts increased pressure. Whereas this model is a helpful analogy, it does not provide mechanistic insight. At a molecular level, the outlet represents t-SNARE complexes comprised of SNAP23 and an unknown Syntaxin [4,5]. The hydrostatic pressure represents secretory granules, which could restore mucin release either by increasing in number or size, probably in association with redistribution of the v-SNARE, VAMP8 [13], from a reserve compartment to secretory granules where they are positioned for productive exocytic *trans*-SNARE pairing. These two possibilities cannot be distinguished at the present time because mucin granules cannot be clearly identified by electron microscopy in naive WT mice [10].

**Figure 7:**
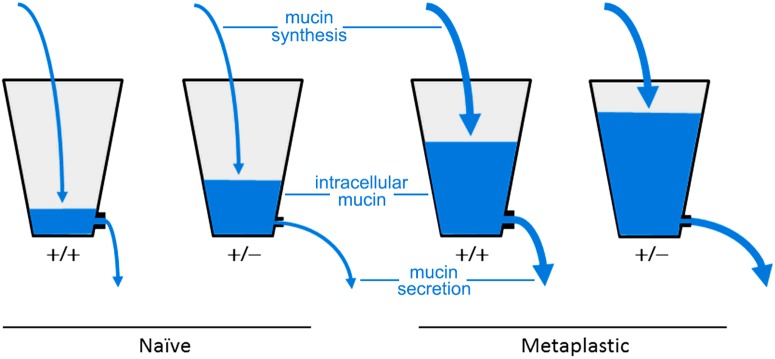
Hydrostatic steady state model of the effects of SNAP23 mutation on mucin accumulation and baseline secretion The naive uninflamed condition with low mucin synthesis is illustrated with thin arrows of water flowing into the two left buckets, whereas the metaplastic inflamed condition with high mucin synthesis is illustrated with thick arrows flowing into the two right buckets. Buckets representing WT (+/+) mice have large outlets so that little water accumulates in the naive condition and only a moderate amount in the metaplastic condition, whereas buckets representing SNAP23 Het (+/−) mice have small outlets so that some water accumulates even in the naive condition and a large amount in the metaplastic condition. In all scenarios, hydrostatic pressure increases until steady state outflow matches inflow.

For stimulated secretion, a static assay of intracellular mucin content (image analysis in Figure 3B) shows impaired release in SNAP23 Het mice that is roughly commensurate with the level of SNAP23 protein (Figure 6A). However the kinetic assay of released mucins shows a defect in stimulated secretion in Het mice only during the first 10 min after stimulation, whereas mucin release during the ensuing 45 min of stimulation is actually higher in Het than in WT mice (Figure 5). In fact, these two different assays are consistent because the static assay (Figure 3B) reflects just 5 min of stimulation (see ‘Materials and Methods’). Assuming that the amount of released mucin reflects the product of release efficiency and intracellular store size as for baseline secretion, the defect in release efficiency in SNAP23 Het mice dominates during the first 5–10 min of stimulation whereas the larger mucin store dominates subsequently. This probably reflects the inability of increased mucin stores to fully compensate for reduced release efficiency during high initial rates of stimulated mucin release, consistent with the profound depletion of mucin stores after just 5 min of stimulation in WT mice in the static assay (Figure 3B). This interpretation is supported by high resolution *in vitro* kinetic studies of canine and human airways that showed a burst of exocytosis within 10 s of exposure to ATP with a rate several 1000-fold greater than at baseline, followed by a plateau lasting several minutes with a rate ~10-fold greater than at baseline and with most cells depleted of granules after 5 min of ATP exposure [44,45].

### Functional consequences of the partial defect in mucin secretion

Even though the constitutively produced airway mucin Muc5b is essential for particle clearance and defence against bacteria [3], there were no apparent abnormalities in the clearance of particles or bacteria from the lungs of SNAP23 Het mice (Figure 6B). This can be explained because clearance functions were measured under conditions of baseline mucin secretion, which is normal in SNAP23 Het mice despite the defect in exocytic function (Figure 7). Probably for the same reason, airway development appears to be normal (Figure 6C). We are unable to determine whether there are negative functional consequences of the kinetic defect in stimulated mucin secretion in SNAP23 Het mice because the physiologic role of stimulated mucin secretion is not known. It has been hypothesized that mucin secretion may be stimulated locally to promote pathogen or particle clearance [1,2] or to cause airway occlusion to prevent helminthes that migrate through the lungs from ascending airways to be swallowed to complete their life cycles [46–48]. Pathogen challenges of mice with a more severe defect in stimulated secretion could test these hypotheses.

Pathophysiologically, widespread and sudden stimulation of mucin secretion causes airflow obstruction in asthma [1,47]. This occurs when inhaled allergens or irritants induce the release of mucin secretagogues such as histamine from mast cells or acetylcholine from neurons, which in turn cause the release of mucin stores from metaplastic epithelium. Blunting the acute phase of stimulated mucin secretion in this setting might prevent formation of concentrated mucus that cannot be cleared by ciliary action and therefore contributes to airflow obstruction [1,49,50,51]. We were unable to demonstrate such benefit (Figure 6E), possibly because the defect in stimulated secretion in SNAP23 Het mice is insufficiently severe or our conventional experimental paradigm of escalating doses of MCh resulted in gradual depletion of intracellular mucin stores rather than sudden catastrophic release. Future studies of the therapeutic manipulation of airway mucin secretion using mice with more severe defects in stimulated secretion and an acute paradigm of MCh exposure could resolve these questions.

### Summary

SNAP23 is selectively expressed in secretory cells of the airway epithelium, where it mediates both baseline and stimulated mucin secretion. The baseline mucin secretory rate of SNAP23 Het mice is normal but the acute phase of the stimulated rate is reduced, suggesting that increased intracellular mucin stores can compensate for reduced exocytic efficiency at low intensities of stimulation. Analysis of homozygous cellular deletion of SNAP23 in airway secretory cells will be required to determine whether SNAP23 mediates all non-Syntaxin t-SNARE function in baseline and stimulated mucin secretion and what the functional consequences are of its complete loss.

## Abbreviations

CCSP: club cell secretory protein/secretoglobin 1A1
DMEM: Dulbecco’s modified Eagle’s medium
GAPDH: glyceraldehyde 3-phosphate dehydrogenase
HRP: horseradish peroxidase
Het: heterozygous gene deletant
KO: homozygous gene deletant
MCh: methacholine
Muc5ac and Muc5b: secreted mucin glycoproteins encoded by distinct genes
Munc13: mammalian orthologues of the *C. elegans* unc-13 protein uncovered in a screen for uncoordinated mutants
Munc18: mammalian orthologues of the *C. elegans* unc-18 protein
OPT: optical projection tomography
PAFS: periodic acid fluorescent Schiff’s reagent
PBST: PBS containing 0.05% Tween-20
PFA: paraformaldehyde
SM protein: a protein of the extended Sec1/Munc18 family, comprised in mammals of three Munc18 proteins, Sec1, Vps33a, Vps33b and Vps45
SNAP23: 23-kDa paralogue of synaptosome-associated protein of 25 kDa
SNARE: soluble NSF attachment protein receptor
Syt: synaptotagmin
t-SNARE: target membrane SNARE protein
VAMP: vesicle-associated membrane protein
v-SNARE: vesicle membrane SNARE protein
WT: wild-type
